# Research on Economic Load Dispatch Problem of Microgrid Based on an Improved Pelican Optimization Algorithm

**DOI:** 10.3390/biomimetics9050277

**Published:** 2024-05-04

**Authors:** Yi Zhang, Haoxue Li

**Affiliations:** College of Electrical and Computer Science, Jilin Jianzhu University, Changchun 130119, China

**Keywords:** economic load dispatch, pelican optimization algorithm, crisscross optimization algorithm, dimensional variation strategy

## Abstract

This paper presents an improved pelican optimization algorithm (IPOA) to solve the economic load dispatch problem. The vertical crossover operator in the crisscross optimization algorithm is integrated to expand the diversity of the population in the local search phase. The optimal individual is also introduced to enhance its ability to guide the whole population and add disturbance factors to enhance its ability to jump out of the local optimal. The dimensional variation strategy is adopted to improve the optimal individual and speed up the algorithm’s convergence. The results of the IPOA showed that coal consumption was reduced by 0.0292%, 2.7273%, and 3.6739%, respectively, when tested on 10, 40, and 80-dimensional thermal power plant units compared to POA. The IPOA can significantly reduce the fuel cost of power plants.

## 1. Introduction

The economic load dispatch (ELD) problem is a fundamental problem in power system control and operation [1]. The goal of ELD is to find the best feasible power generation plan with the lowest fuel cost to meet the generation constraints of the generator set [2]. The power generation system must also comply with various practical limitations due to the physical constraints associated with the system in addition to meeting the system’s power needs. These limitations result in the ELD problem being a non-convex, non-continuous, non-differentiable optimization problem with many equality and inequality constraints [3].

There is much literature on the ELD problem, proposing many methods. These algorithms can be mainly divided into two categories. One is traditional optimization methods, such as the gradient method [4], Lambda iterative method [5], and quadratic programming method [6]. These methods may not converge to a feasible solution in the solving process [7,8], and it is not easy to get a satisfactory solution in an adequate time [9]. The second category is intelligent optimization algorithms inspired by nature’s physical or biological behavior [10], which have the characteristics of flexible mechanisms, simple operation, and efficient solutions [11,12]. They have advantages in solving large-scale and highly complex optimization problems [13,14]. Many swarms’ intelligent optimization algorithms have recently been applied to solve the ELD problem [15]. Arman Goudarzi et al. [16] proposed a new algorithm, MGAIPSO, based on an improved genetic algorithm and a version of particle swarm optimization. Namrata Chopra et al. [17] proposed an improved particle swarm optimization algorithm using the simplex method. Seyedgarmroudi, S.D. et al. [18] proposed an improved pelican optimization algorithm, which benefited from three motion strategies, predefined knowledge-sharing factors, and a modified dimension learning-based hunting (DLH). Singh, N. et al. [19] utilized a new variant of particle swarm optimization. Lotfi, H. et al. [20] proposed an improved modified grasshopper optimization algorithm, based on the chaos mechanism. Ismaeel, A.A.K. et al. [21] used the osprey optimization algorithm. Said, M. et al. [22] utilized the walrus optimizer. Almalaq, A. et al. [23] introduced a new multi-objective optimization technique combining the differential evolution (DE) algorithm and chaos theory. Dey, B. et al. [24] proposed a new optimization algorithm combining the greedy JAYA algorithm with an algorithm based on a crow’s food-seeking approach. Acharya, S. et al. [25] proposed the multi-objective multi-verse optimization (MOMVO) algorithm. These algorithms have been applied to solve ELD problems and have achieved good results. However, there is still room for further improvement in the quality and applicability of these algorithms. Therefore, to solve the ELD problem more effectively, exploring the algorithm with better optimization ability, higher solution accuracy, and more stable solution results is necessary.

Many excellent meta-heuristics have been proposed in recent years, such as the liver cancer algorithm (LCA) [26], slime mould algorithm (SMA) [27], moth search algorithm (MSA) [28], parrot optimizer (PO) [29], rime optimization algorithm (RIME) [30], and pelican optimization algorithm (POA) [31]. The POA is a new meta-heuristic intelligence algorithm proposed by Pavel Trojovský et al. in 2022. The algorithm has the characteristics of simple theory, easy implementation, and good solving performance, and is suitable for solving large-scale complex optimization problems, including ELD problems [32]. Therefore, many scholars have conducted in-depth research and applied it to different fields [33]. For example, Song, H.M. et al. [34] proposed an improved POA based on chaotic interference factors and essential mathematical functions and applied it to four engineering design problems. Eluri, R.K. et al. [35] proposed a chaotic binary search gecko optimization algorithm. By converting the basic algorithm into binary and chaotic search and enhancing the POA’s exploration and development process, Li, J. et al. [36] used elite reverse learning, introduced Levy flight to improve the POA, and applied it to microgrid scheduling. Xiong, Q. et al. [37] improved the POA by introducing fractional order chaotic sequence and applied it to the memo chaotic system parameter identification. Tuerxun et al. [38] optimized the generalized learning system’s parameters by improving the POA. Abdelhamid, M. et al. [39] proposed an improved pelican optimization algorithm and applied it to the protection of distributed generators. Chen, X. et al. [40] used the pelican optimization algorithm (POA) to optimize the neural network prediction model, which significantly improved the model’s accuracy. Zhang, C. et al. [41] proposed a symmetric cross-entropy multilevel threshold image segmentation method (MSIPOA) with a multi-strategy improved pelican optimization algorithm for global optimization and image segmentation tasks. 

The above improvements have enhanced the application capability of the POA in their respective fields. However, according to the NFL (no free lunch) [42] theorem, there is no single algorithm that can solve all optimization problems [43]. Therefore, there is still room for further enhancement of the stability of the POA and its suitability for large-scale complex applications [44]. 

This paper proposes an improved pelican optimization algorithm (IPOA) to solve the ELD problem and improve the POA’s search performance and quality. This IPOA utilizes the crisscross optimization algorithm and introduces disturbance factors and dimensional variation strategy. First, in the local search phase, the crisscross optimization algorithm is integrated to expand the diversity of the population. After that, the optimal individual is introduced to enhance the guiding ability, accelerate the convergence speed, and add a disturbance factor to enhance the ability to jump out of the local optimal. Thirdly, the dimensional variation strategy is adopted to improve the optimal individual and speed up the algorithm’s convergence. In this paper, the effectiveness of the IPOA is tested on eight CEC2017 test functions. The experimental results show that the optimization performance and quality of the IPOA are better than those of the other four algorithms. At the same time, the IPOA is used to solve the ELD problem. It is applied in different units of 10, 40, and 80 dimensions, respectively. The experimental results show that the IPOA has good optimization ability and reliability and can effectively solve the problem of the high operating costs of power systems.

The structure of this paper is as follows: Section 2 establishes the mathematical model of the ELD and introduces the pelican optimization algorithm, including its improved version (IPOA), which is subsequently tested on the CEC2017 test functions, with the results analyzed. In Section 3, the IPOA is applied to the ELD problem with 10, 40, and 80 units, and its ability to solve practical problems is tested. Section 4 then summarizes the findings.

## 2. Materials and Methods

### 2.1. Relationship Work

#### 2.1.1. Electric Power Economic Load Dispatch (ELD)

The problem of electric power economic load dispatch (ELD) is an important power system optimization problem at present. Minimizing the cost is the objective under the premise of satisfying the equation and inequality constraints. The following objectives and constraints were considered in the formulation of this paper. The objective function in the ELD problem can be expressed as:

(1) $$Min\sum_{i=1}^{N}F_{i}{(p}_{i})$$

In Equation (1), *N* is the total number of generator sets, $F_{i}$ is the fuel cost function of the *i*_th_ generator set, and $p_{i}$ is the generation capacity of the *i*_th_ generator set according to the generation plan. The generator’s cost function is derived from data points acquired during the “hot run” test. The opening of the steam intake valve changes discontinuously when the load is adjusted in the thermal generator set. It will cause the efficiency and cost of the unit to fluctuate. This phenomenon is known as the valve point effect, and it stops the cost curve from being smooth. Therefore, the valve point effect must be included in the cost model to represent the power generation cost curve more accurately. Therefore, the actual output power of the total fuel cost can be expressed as [45]:

(2) $$F\left(p_{i}\right)=a_{i}p_{i}^{2}+b_{i}p_{i}+c_{i}+|e_{i}*sin\left\{f_{i}*\right.\left.\left(p_{i}^{min}-p_{i}\right)\right\}|$$

In Equation (2), $F_{i}\left(p_{i}\right)$ represents the fuel cost function of the *i*_th_ unit, and $p_{i}$ represents the generation capacity of the *i*_th_ unit according to the generation plan. The parameters *a*, *b*, and *c* are constants determined by the physical characteristics of the unit, the parameters *e* and *f* are coefficients describing the valve point effect, and $p_{i}^{min}$ represents the minimum power output of the *i*_th_ unit. 

The capacity constraints must be met to ensure the safe operation of thermal power units; the formula is as follows:

(3) $$p_{i}^{min}\leq p_{i}\leq p_{i}^{max}$$

In Equation (3), $p_{i}^{min}$ and $p_{i}^{max}$ represent the minimum and maximum power output of the *i*_th_ power unit, respectively. The sum of power of each unit needs to be consistent with the total load because power transmission loss is ignored in this paper, and the load balance constraint formula is as follows:

(4) $$\sum_{i=1}^{N}p_{i}=p_{d}$$

In Equation (4), $p_{d}$ represents the load demand.

This paper presents a penalty mechanism method to deal with the constraints in the ELD problem to balance the objective function and constraints and transform the constrained problem into an unconstrained problem. The solution in the optimization process is forced to meet all constraints by the introduction of a penalty term into the objective function. The objective function after the introduction of the penalty term can be described as:

(5) $$Min(\sum_{i=1}^{N}f_{i}{(p}_{i})+\varepsilon *|\sum_{i=1}^{N}p_{i}-p_{d}|)$$

In Equation (5), $\sum_{i=1}^{N}p_{i}$ represents the total generating capacity of all units according to the power generation plan, and ε is the penalty function coefficient.

#### 2.1.2. Pelican Optimization Algorithm

The pelican optimization algorithm is a natural heuristic algorithm proposed by Pavel Trojovský et al. in 2022 [31]. The model simulates pelicans’ hunting behavior. It can be divided into two stages: approaching prey (exploration stage) and surface flight (development stage).

**Population initialization**

Before hunting, it is necessary to initialize the pelican population, where each member represents a candidate solution represented by a vector. The mathematical model is shown in Equation (6):

(6) $$X_{i,j}=l_{j}+rand*\left(u_{j}-l_{j}\right),\;\;\;\;i=1,2,\ldots ,\;N,\;\;\;\;j=1,2\ldots ,m$$

In Equation (6), $X_{i,j}$ represents the position of the *i*_th_ pelican in the *j* dimension, *N* is the population number of pelicans, *m* is the dimension of the problem, and *rand* represents the random number [0, 1]. $u_{j}$ and $l_{j}$ represent the upper and lower bounds of the *J*_th_ dimension of the problem, respectively.

**Exploration phase**

In the first stage, the prey positions are randomly generated within the search space, and the pelicans determine the prey positions. If the objective function value of the pelicans is less than that of the prey, they move towards the prey; otherwise, they move away from the prey. Its mathematical model is shown in Equation (7):

(7) $$X_{i}^{P_{1}}=\left\{\begin{array}{l} X_{i}+rand*(P-I*X_{i}),\;\;F_{p}<F_{i}\\ X_{i}+rand*\left(X_{i}-P\right)\;\;\;\;\;,\;\;else \end{array}\right.$$

In Equation (7), $X_{i}^{P_{1}}$ represents the position of the *i*_th_ pelican after the first stage update, *I* represent 1 or 2 random integers, *P* represents the position of the prey, *rand* represents the random number [0, 1], $F_{p}$ represents the fitness value of the prey, and $F_{i}$ represents the fitness value of the *i*_th_ pelican.

The pelican updates its position if the fitness value of the new position is better than the previous position after the pelican moves toward the prey. Its mathematical model is shown in Equation (8):

(8) $$X_{i}=\left\{\begin{array}{l} X_{i}^{new},\;\;F_{i}^{new}<F_{i}\\ X_{i}\;\;\;,\;\;else \end{array}\right.$$

In Equation (8), $X_{i}^{new}$ represents the updated position of the *i*_th_ pelican, and $F_{i}^{new}$ represents the fitness value of the updated new position.

**Development phase**

In the second stage, after the exploration stage is completed, the pelicans enter the exploitation stage. Upon reaching the water surface, the pelicans capture the prey. During this stage, the algorithm searches for points within the neighborhood of the pelican’s position to achieve better convergence. Its mathematical model is shown in Equation (9):

(9) $$X_{i}^{P_{2}}=X_{i}+R*\left(1-\frac{t}{T}\right)*\left(2*rand-1\right)*X_{i}$$

In Equation (9), $X_{i}^{P_{2}}$ represents the position of the *i*_th_ pelican after the second stage update, *R* is the constant 0.2, *rand* represents the random number [0, 1], and *t* and *T* represent the current and maximum iterations, respectively. 

In the development phase, the location is updated if the fitness value of the new location is better than the location before the move after the pelican location is updated as in the exploration phase. If not, it is left in place.

### 2.2. Improved Pelican Optimization Algorithm

In this paper, three strategies were introduced to improve the accuracy, convergence speed, and robustness of the POA.

#### 2.2.1. Fusion of Improved Crisscross Optimization Algorithm for Local Search

Crisscross optimization algorithm (CSO) [46] is a new search algorithm proposed by An-bo Meng et al. in 2014. The CSO uses vertical and horizontal crosses to update the position of individuals in a population, inspired by the cross operation in the Confucian mean and genetic algorithm. The horizontal crossing is the arithmetic crossing of all dimensions between two different individuals, whose calculation formula is:

(10) $${MS}_{hc}\left(i,d\right)=r_{1}*X\left(i,d\right)+\left(1-r_{1}\right)*X(j,d)+C_{1}*(X(i,d)-X(j,d))$$

(11) $${MS}_{hc}\left(j,d\right)=r_{2}*X\left(j,d\right)+\left(1-r_{2}\right)*X(i,d)+C_{2}*(X(j,d)-X(i,d))$$

In Equations (10) and (11), $X\left(i,d\right)$ and $X\left(j,d\right)$ represent the positions of the *d* dimension of the *i*_th_ and *j* individuals, respectively; $r_{1}$ and $r_{2}$ represent the random numbers between 0 and 1; and $C_{1}$ and $C_{2}$ represent the random numbers between −1 and 1. ${MS}_{hc}\left(i,d\right)$ and ${MS}_{hc}\left(j,d\right)$ represent the offspring produced after horizontal crossing.

A vertical crossover is an arithmetic crossover that operates on all individuals between two different dimensions, calculated by:

(12) $${MS}_{vc}\left(i,d1\right)=r*X\left(i,d_{1}\right)+\left(1-r\right)*X(i,d_{2})$$

In Equation (12), $X\left(i,d_{1}\right)$ and $\;X\left(i,d_{2}\right)$ represent the positions of the $d_{1}$ and $d_{2}$ dimensions of the *i*_th_ individual respectively, *r* represents the random number between 0 and 1, and ${MS}_{vc}\left(i,d1\right)$ represents the offspring produced after vertical crossing.

The POA easily falls into the local optimal because the pelican individual moves within a small range in the local search process. The CSO is integrated into the local search stage of the POA to enhance its ability to jump out of the local optimal because of strong global detection ability and local development ability. In the original POA, the current individual will be far away from the randomly generated individual when the fitness value of the randomly generated individual is less than that of the current individual. The randomly generated individuals are not fully utilized. In this paper, the horizontal crossover in the CSO is introduced to make full use of the random individuals, guide the pelican individuals to move to the target position, and enhance the local development ability of the algorithm and its ability to jump out of the local optimal. The improved formula is as follows:

(13) $$X_{i}^{p_{1}}(i,j)=r_{1}*X(i,j)+\left(1-r_{1}\right)*P(i,j)+\sin\left(r_{2}\right)*(X(i,j)-P(i,j))$$

In Equation (13), $X\left(i,j\right)$ represents the current individual; P(i,j) represents the random individual, i.e., the prey; $r_{1}$ represents the random number between 0 and 1; and $r_{2}$ represents the random number between 0 and 2π.

#### 2.2.2. Improved Global Search

The pelicans only use their current position to update their positions according to the POA principle in the global search stage. The position of the optimal individual is not fully utilized, which makes the development ability of the algorithm insufficient. This paper introduces the optimal individual in the global search stage of the POA to enhance the guidance ability of the overall optimization and increase the ability of the algorithm. At the same time, the adaptive disturbance factor G is introduced to avoid falling into local optimization, and the improved formula is as follows:

(14) $$X_{i}^{P_{2}}={QF*X}_{i}+*\left(2*rand-1\right)*{(X_{best}-X}_{i})+sin(r)*G$$

(15) $$QF\left(t\right)=\frac{2*rand-1}{t^{{\left(1-T\right)}^{2}}}$$

(16) $$G=2*(1-\frac{t}{T})$$

In Equations (14), (15), and (16), *QF* represents the quality function of the balanced search strategy [47], *r* represents the random number from 0 to 2π, *rand* represents the random number [0, 1], and *t* and *T* represent the current and maximum iterations, respectively.

#### 2.2.3. Dimensional Variation Strategy

Like other swarm intelligence algorithms, the POA is prone to local optimality and slow convergence. The analysis shows that the main reason is that the algorithm does not make full use of the guiding role of the optimal individual. Therefore, this paper improves the population diversity by mutating the optimal individual and guiding the population to evolve to the optimal position to improve its convergence speed. At the same time, the strategy of dimensional-by-dimension variation is adopted to update the optimal individual to avoid the problem of inter-dimensional interference in the case of high dimensions. The calculation formula is as follows:

(17) $$X_{new}^{d}=X_{best}^{d}+{TD(t)}^{d}*rand$$

In Equation (17), $X_{new}^{d}$ represents the position of the optimal individual in the *D*-dimension after updating, $X_{best}^{d}$ represents the position of the optimal individual in the *D*-dimension, and *TD*(*t*) represents the *T*-distribution with *t* degrees of freedom [48]. *t* is 25 in this paper. ${TD(t)}^{d}$ represents the random number generated by *t*-distribution in the *D* dimension. To improve the convergence speed, this paper uses the greedy principle to choose whether to use the new position instead of the original optimal position. The specific process is demonstrated in Algorithm 1.
**Algorithm 1**. Mutates Dimensionally1: Generate d random numbers of T-distribution with 25 degrees of freedom parameter. 2: for i = 1: d $3:\;\;\;\;\mathrm{The}\;\mathrm{new}\;\mathrm{solution}\;\mathrm{is}\;\mathrm{obtained}\;\mathrm{after}\;\mathrm{calculating}\;\mathrm{the}\;\mathrm{variation}\;\mathrm{according}\;\mathrm{to}\;\mathrm{Equation}\;(17)\;X_{new}^{d}$ 4:       boundary condition procedure 5:       if fnew < fbest $6:\;\;\;\;\mathrm{Replace}\;\mathrm{the}\;\mathrm{original}\;X_{best\;}^{d}$$\mathrm{with}\;\mathrm{the}\;\mathrm{new}\;\mathrm{position}\;X_{new}^{d}$ $7:\;\;\;\;\mathrm{Calculate}\;\mathrm{the}\;\mathrm{fitness}\;\mathrm{value}\;\mathrm{based}\;\mathrm{on}\;\mathrm{the}\;\mathrm{new}\;\mathrm{position}X_{best}$ 8:   end if 9: end for 10: Return the best fitness value and the best individual

#### 2.2.4. IPOA Implementation Process

**Figure 1:**
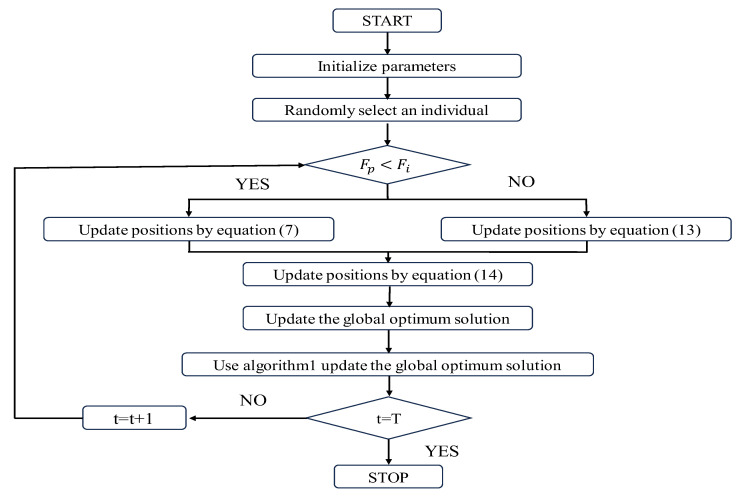
Flowchart of the IPOA.

The specific implementation flowchart of the IPOA is shown in Figure 1, based on the description of the POA improvements in Section 2.1, Section 2.2, Section 2.3.

### 2.3. IPOA Algorithm Performance Test and Analysis

#### 2.3.1. Experimental Environment and Test Function

Simulation environment: 64-bit Windows 10 operating system, processor Intel(R) Core (TM) i5-8265U, main frequency 1.80 GHz, memory 8 GB, programming software MATLAB R2023b. This paper uses CEC2017 test functions to verify the algorithm. The test functions are shown in Table 1, where $f_{1}$ is a unimodal function, $f_{2}$–$f_{4}$ are simple multimodal functions, $f_{5}$ and $f_{6}$ are mixed mode functions, and $f_{7}\;and\;f_{8}$ are combined functions. The algorithm conducted 30 independent experiments on each test function to reduce the randomness and contingency of the algorithm.

#### 2.3.2. Comparisons with POA, PSO, SSA, and WOA

**Figure 2:**
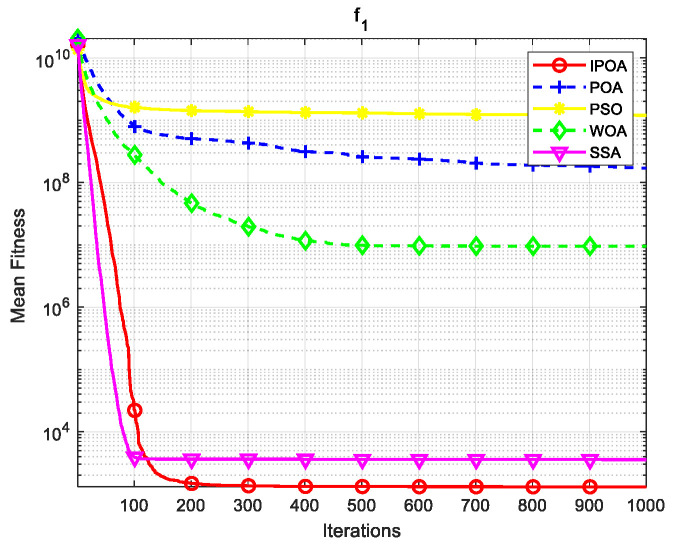
$f_{1}$ iteration diagram.

**Figure 3:**
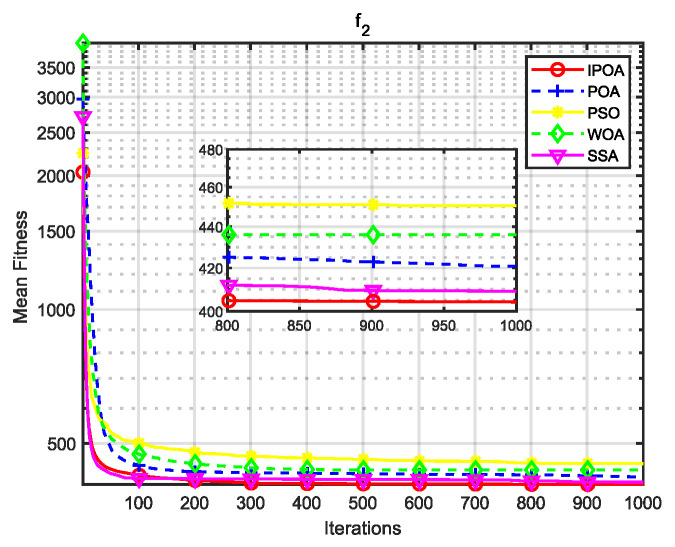
$f_{2}$ iteration diagram.

**Figure 4:**
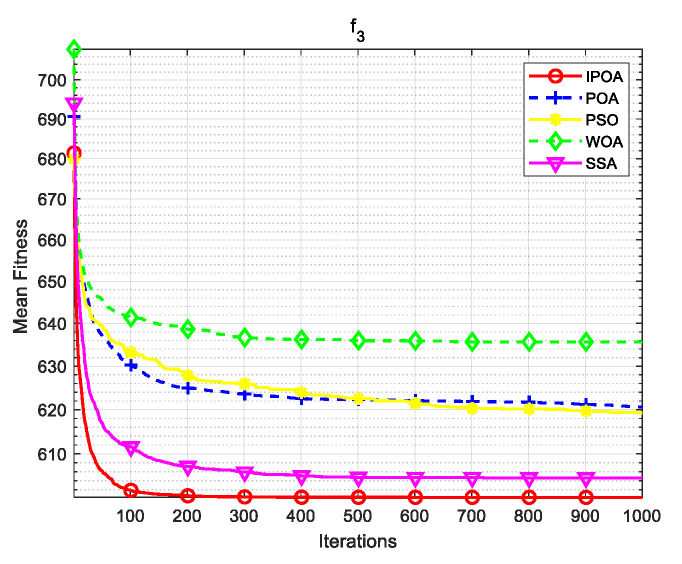
$f_{3}$ iteration diagram.

**Figure 5:**
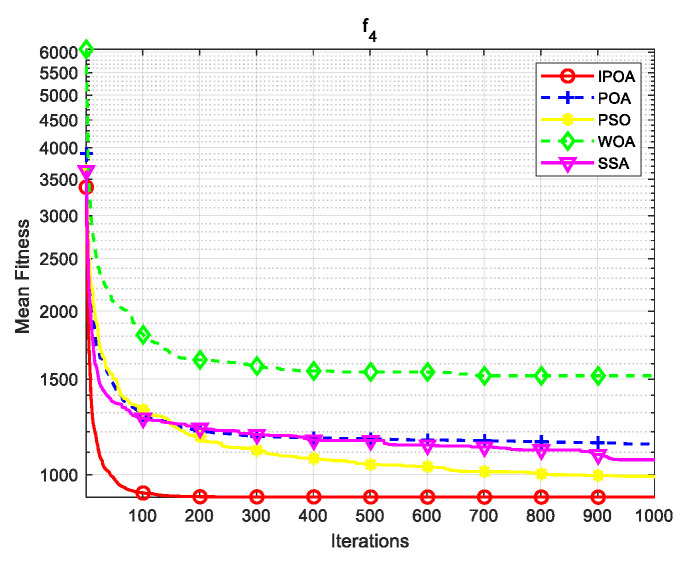
$f_{4}$ iteration diagram.

**Figure 6:**
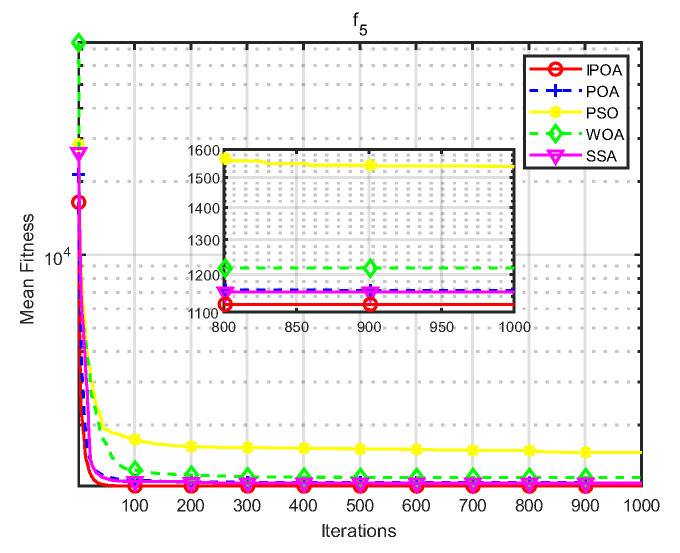
$f_{5}$ iteration diagram.

**Figure 7:**
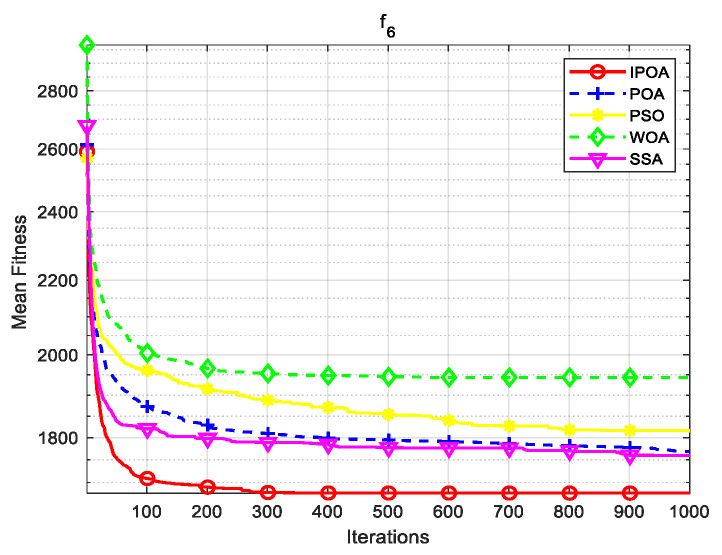
$f_{6}$ iteration diagram.

**Figure 8:**
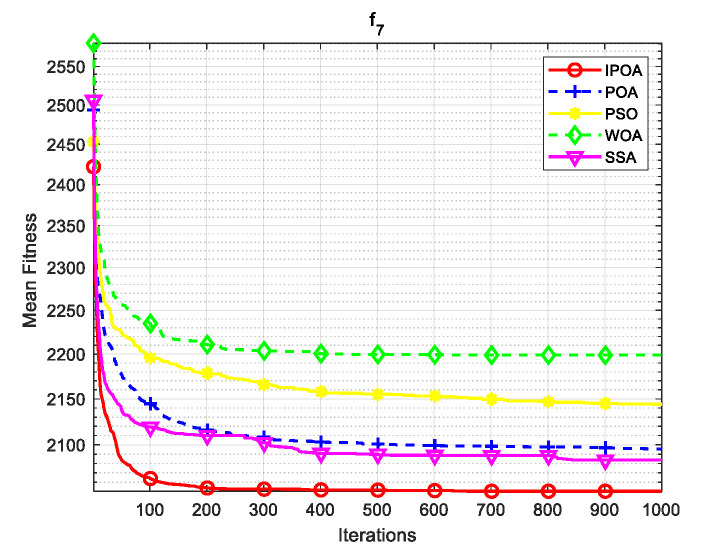
$f_{7}$ iteration diagram.

**Figure 9:**
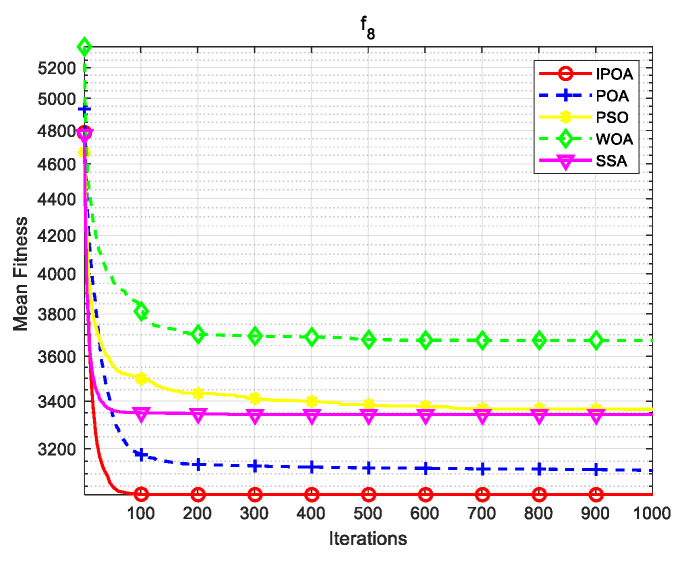
$f_{8}$ iteration diagram.

Four algorithms were selected for comparison with the IPOA to validate its effectiveness. First is the particle swarm optimization algorithm (PSO) [49], which is a classic optimization method, serving as a cornerstone of optimization techniques, and has been widely applied across various domains since its inception. Its performance in both convergence speed and accuracy is exceptional. Additionally, the IPOA is compared with the original pelican optimization algorithm (POA), the sparrow search algorithm (SSA) [50], and the whale algorithm (WOA) [51]. The algorithm parameters were set to the same values as those in the original literature to ensure the fairness of the comparison. The population was 30, and the maximum number of iterations was 1000. The optimization performance of the five algorithms were compared in four respects: best value, worst value, average value, and standard deviation (see Table 2). The convergence curves of each algorithm on the test function are shown in Figure 2, Figure 3, Figure 4, Figure 5, Figure 6, Figure 7, Figure 8 and Figure 9.

The optimization results of the IPOA in eight different tests are superior to those of the POA, PSO, SSA, and WOA, according to the experimental results in Table 2. The IPOA can simultaneously find the theoretical optimal values of the functions $f_{3}$, $f_{4}$, and $f_{7}$, respectively. It is very close to the theoretical optimal values when compared with the other algorithms. Among them, $f_{1}$ is a unimodal function with no local minimum and only a global minimum. In comparison with other algorithms, the IPOA demonstrates significant advantages. As shown in Figure 2, both the SSA and the IPOA exhibit fast convergence speeds, but the IPOA achieves higher accuracy, indicating its strong global convergence capability. $f_{2}$, $f_{3}$, and $f_{4}$ are multi-modal functions with local minimums. The standard deviation of the IPOA is more stable, though the IPOA and the SSA can both find optimal values at $f_{2}$, $f_{3}$, and $f_{4}$ in Table 2. From Figure 4 and Figure 5, it can be observed that the IPOA not only can find the optimal solution but also has a faster convergence speed. From Figure 3, it can be seen that the convergence speed and accuracy of these algorithms are very close, but the IPOA has higher accuracy. This reflects the IPOA’s stronger ability to escape the local optimal compared to the other algorithms. $f_{5}$ and $f_{6}$ are mixed functions of random subfunctions. The hybrid function comprises three or more CEC2017 reference functions, rotated and shifted. Each subfunction is assigned a corresponding weight, which increases the difficulty of the algorithm in finding the optimal solution. $f_{7}$ and $f_{8}$ are composite functions consisting of at least three mixed functions or CEC2017 reference functions after rotation and shifting. Each subfunction has an additional deviation value and is then assigned a weight. These combined functions further increase the optimization difficulty of the algorithm. From Figure 5, Figure 6, Figure 7, Figure 8, Figure 9, it can be seen that the IPOA converges significantly faster compared to the other four algorithms, and the accuracy of the solutions is also higher. Whether observed horizontally or vertically, the IPOA outperforms the other four algorithms, indicating its strong convergence performance and excellent exploration ability. 

The improvements incorporated the crisscross optimization algorithm in the local search stage to improve the diversity of the population; at the same time, the optimal individual is introduced in the global search stage to enhance the guiding ability of the whole population, and the disturbance factor is added to increase the ability to jump out of the local optimal; finally, the optimal individual is adopted by the dimensional-by-dimension variation strategy to guide the evolution to the optimal position better, thereby improving the convergence speed of the algorithm, leading to better performance of the IPOA compared to the other algorithms. 

### 2.4. IPOA Solves the Problem of Economic Dispatch

Firstly, the relevant parameters of the IPOA algorithm need to be adjusted in the process of solving the ELD problem. The population is randomly generated with the upper and lower limits of the power load as constraints, and the population represents the unit output. The objective function proposed after considering the penalty coefficient is taken as the fitness function, and the number of units is taken as the solution dimension. Secondly, the IPOA is used to update the population and find the individual that can minimize the fitness function. Then, Formula (1) is used to calculate the minimum cost. Finally, the optimal load distribution and coal consumption of each unit are outputted. The specific process is demonstrated in Algorithm 2.
**Algorithm 2**. IPOA for ELD1: **Input:** Population size, Dimension, variable bounds Maximum failure count 2: **Initialization:** Initialize population X and Calculate fitness value using Equation (5) 3: for i = 1: Max_iterations 4:       for j = 1: N 5:         Randomly select an individual 6:         if fit(p) < fit(j) 7:       Update positions by Equation (7) 8:         else 9:       Update positions by Equation (13) 10:        end if 11:        Update positions by Equation (14) 12:        Use algorithm1 update the global optimum solution 13:        Handling boundary conditions 14:        Calculating individual fitness values using Equation (5) 15:        Update the global optimum solution 16:   end for 17: end for 18: Calculate fuel cost using Equation (1) 19: **Output:** Optimal cost, Unit’s output

## 3. Experimental Results and Discussion

In this paper, 10 small power plants and 40 medium power plants were selected to evaluate the effectiveness of the IPOA algorithm. The test results of the IPOA were compared with those of the POA, the Harris hawk’s optimization (HHO) [52], the SSA, and the WOA to verify the solving ability of the IPOA more comprehensively. The parameters of the algorithm were set to the same values as in their respective original literature in order to ensure the fairness of comparison, and the number of runs, population size, spatial dimension, and maximum number of iterations were made consistent. That is, the population was 30, the maximum number of iterations was 1000, and the algorithm was run independently 30 times.

### 3.1. 10 Units

In this case study, the ELD system was composed of 10 generator sets; the coal consumption characteristic parameters of the unit and the upper and lower limits of the unit load [53] are shown in Table 3. Unit data for the 10 generating units power system with VPE. The total load demand was 2700 MW.

**Figure 10:**
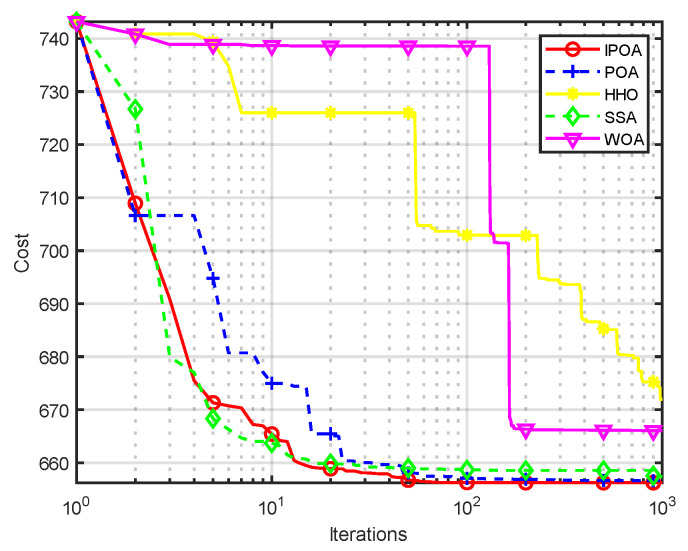
Convergence curve of unit 10.

Different algorithms cannot generate feasible solutions meeting the constraint conditions simultaneously because of the same penalty function coefficient. Different penalty function coefficients were applied to the different algorithms based on the experimental results. For the IPOA it was 0.500, for the SSA, WOA, HHO it was 0.531, and for the POA it was 0.610. After 30 independent experiments of each algorithm, the optimal output of each unit is shown in Table 4. The total fuel cost of the IPOA was the lowest at 651.8784 ($/h), as seen in Table 5. Compared with the traditional POA algorithm, the coal consumption was reduced by 0.1903 ($/h), a decrease of 0.0292%. Compared with the whale algorithm (WOA), the coal consumption was reduced by 1.6003 ($/h), a decrease of 0.2455%. And it can be seen from Figure 10 that the IPOA demonstrated faster convergence speed and better convergence results. Reducing total fuel cost can improve the efficiency of a power plant and reduce its economic costs. Compared with the other four algorithms, the standard deviation of the IPOA was the smallest, which indicates that the improved pelican optimization algorithm has good development ability and strong stability in dealing with ELD problems.

### 3.2. 40 Units

In this section, a medium-sized power plant of 40 units is taken as an example, with a load demand of 10,500 MW. The coal consumption characteristic parameters of the unit and the upper and lower limits of the unit load [54] are shown in Table 6. The penalty function coefficients of the IPOA, WOA and SSA were 17.5, HHO was 16, and POA was 21.5. The optimal output of each unit is shown in Table 7 after 30 independent experiments.

**Figure 11:**
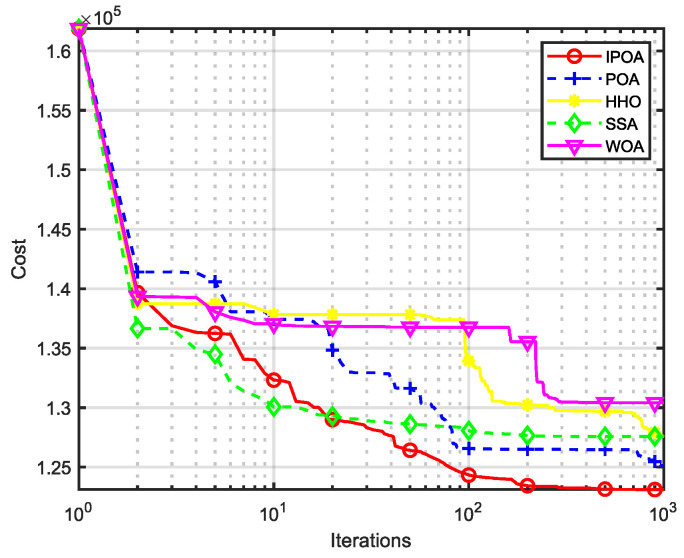
Convergence curve of unit 40.

The total fuel cost of the IPOA was the lowest at 121,591.3068 ($/h), as shown in Table 8. The coal consumption was reduced by 3316.1208 ($/h) compared with the traditional POA algorithm, a decrease of 2.7273%, and the effect was more significant than that of the WOA. The coal consumption decreased by 4288.7396 ($/h), or 3.5272%. The standard deviation of the IPOA was the smallest among the five algorithms, the convergence speed of the IPOA in the early stage was second only to the SSA, as seen in Figure 11, and the convergence speed in the later stage was the fastest, all of which indicate that the IPOA has faster convergence speed and better convergence results. This is mainly because the IPOA adopts a dimensional-by-dimension variation strategy to avoid the problem of inter-dimensional interference in the case of high dimensions, which allows it to perform well in practical problems in high dimensions. The longitudinal crossover strategy was introduced to improve the diversity of the population and the stability of the algorithm. In the local development stage, the optimal individual and disturbance factor were introduced to improve the convergence ability of the algorithm.

### 3.3. 80 Units

In this section, the system was built by repeating the 40-unit system twice, with a load requirement of 21,000 MW, by taking a large power plant with 80 units as an example. The local minima of the solutions increase as the number of solutions increases. Therefore, the solution algorithm needs a stronger global search ability to overcome the precocious convergence problem. The penalty function coefficients of each algorithm were as follows: 17.5 for the IPOA, HHO, WOA and SSA, and 20.5 for the POA. Each algorithm underwent 30 independent experiments.

The total fuel cost of the IPOA was 244,105.2816 ($/h), as shown in Table 9; the coal consumption was reduced by 8968.1651 ($/h) compared with the traditional POA algorithm, a reduction of 3.6739%, and by 2427.0296 ($/h) compared with the second-best sparrow algorithm (SSA), a decrease of 0.9943%. The application results of the IPOA in large units were better than those in small and medium-sized units, indicating that IPOA has significant advantages in dealing with high-dimensional problems.

## 4. Conclusions

**Figure 12:**
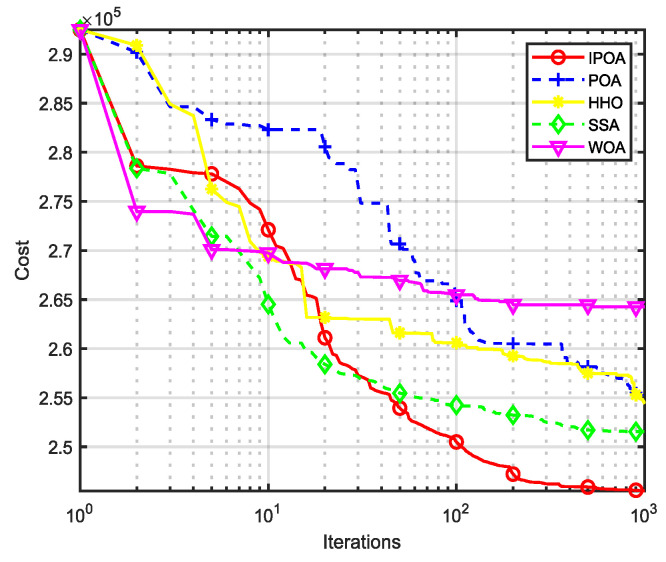
Convergence curve of unit 80.

This paper proposes an improved pelican optimization algorithm (IPOA) to optimize the original POA by using longitudinal crossover and dimensional variation strategies and introducing optimal individuals and disturbance factors in the global phase. In this paper, the IPOA was tested on eight CEC2017 test functions, and the test results show that the algorithm can jump out of the local area. Secondly, the IPOA was applied to the economic scheduling problem of thermal power units with multiple practical constraints, and its effectiveness was verified with 10 units, 40 units, and 80 units, respectively. In the case of low-dimension 10 units, the coal consumption was reduced by 0.0292% compared with the original POA. In the case of 40 units, it was reduced by 2.7273% compared with the original POA. In the case of high-dimension 80 units, it was reduced by 3.6739% compared with the POA; from Figure 12, it can be observed that compared to the cases with 10 units and 40 units, the IPOA exhibited a more significant advantage in both convergence speed and convergence accuracy, indicating that it has a significant advantage in solving high-dimensional problems. The IPOA showed that the improved method has good performance in solving the complex non-convex ELD problem, which can significantly reduce coal consumption and improve the economic benefit of power plants. The algorithm is promising and can be applied to other complex practical problems. In the follow-up study, we will apply the IPOA to the economic scheduling problem of multi-fuel and multi-constrained thermal power units to better verify the algorithm’s performance.

## Figures and Tables

**Table 1:** Test functions.

	Functions	Best Value	Types
$f_{1}$	Shifted and Rotated Bent Cigar	100	Unimodal
$f_{2}$	Shifted and Rotated Rastrigin’s	400	Simple Multimodal
$f_{3}$	Shifted and Rotate Lunacek Bi_Rastrigin	600	
$f_{4}$	Shifted and Rotated Schwefel’s	900	
$f_{5}$	Hybrid Function 2 (N = 3)	1100	Hybrid
$f_{6}$	Hybrid Function 6 (N = 5)	1600	
$f_{7}$	Composition Function 1 (N = 3)	2000	Composition
$f_{8}$	Composition Function 7 (N = 6)	2600	

**Table 2:** Evaluation results of test functions.

Function	Index	Algorithm				
		IPOA	POA	PSO	WOA	SSA
$f_{1}$	Best	**100.7108**	6097	57,929,542	592,934.6706	133.5179
	Worst	1226.4633	1,944,227,684	2,850,258,819	74,471,522.3021	12,381.7875
	Mean	4768.0518	219,781,765	792,680,137	9,361,246.7600	4438.0798
	Std	**1560.1471**	495,103,154	831,763,818	15,616,489.4653	3567.2322
$f_{2}$	Best	**400.0002**	400.7755	411.1083	400.6303	400.1664
	Worst	473.2955	496.9909	825.9439	563.8685	472.0999
	Mean	404.1049	418.4436	505.0500	440.8641	404.6719
	Std	**13.1237**	21.6410	102.3337	47.2803	12.7660
$f_{3}$	Best	**600.0000**	607.4886	608.6856	610.2782	**600.0000**
	Worst	601.4796	638.7122	635.2207	657.3534	613.0539
	Mean	600.1154	621.6511	618.6479	634.4335	602.8292
	Std	**0.33723**	9.2482	6.2520	11.8559	3.8600
$f_{4}$	Best	**900.0000**	906.0017	931.4729	921.8618	**900.0000**
	Worst	929.6692	1387.7745	1293.9060	3566.0554	1829.3862
	Mean	903.8361	1092.0306	1008.2076	1612.5818	1116.2890
	Std	**7.1136**	141.2868	65.9039	643.7356	308.2101
$f_{5}$	Best	**1100.0366**	1109.0200	1171.9598	1123.9527	1103.0719
	Worst	1137.6526	1315.8967	1903.2485	1568.3389	1258.4352
	Mean	1116.7835	1171.1777	1345.1482	1208.2003	1145.7665
	Std	**10.4314**	49.0942	165.8870	90.6109	41.5469
$f_{6}$	Best	**1600.7438**	1607.6178	1636.0000	1622.9391	1601.4464
	Worst	1960.8268	1938.6274	2246.0736	2304.1432	2139.5167
	Mean	1689.9921	1763.1663	1804.9491	1913.4186	1832.8280
	Std	115.7566	**106.2803**	157.1715	187.8456	138.5551
$f_{7}$	Best	**2000.0000**	2024.1393	2057.8897	2043.5482	2005.5991
	Worst	2140.3403	2162.7398	2244.1384	2450.8169	2278.7248
	Mean	2037.2731	2083.7765	2127.0903	2191.1716	2088.4404
	Std	**26.1220**	40.1675	58.2918	88.8603	67.2939
$f_{8}$	Best	**2600.0043**	2608.7520	2967.3881	2628.1123	2800.0000
	Worst	3165.7513	3904.5444	4257.0824	4786.9540	3395.5483
	Mean	2966.1859	3023.9175	3329.9615	3585.4080	4483.6695
	Std	**134.8942**	241.6267	450.6247	574.6367	532.1813

**Table 3:** Unit data for the 10 generating units power system with VPE.

Units	Pmin	Pmax	a	b	c	e	f
1	100	250	0.002176	−0.3975	26.97	0.02697	−3.975
2	50	230	0.004194	−1.269	118.4	0.1184	−12.69
3	200	500	0.00001176	0.4864	−95.14	−0.05914	4.864
4	99	265	0.005935	−2.338	266.8	0.2668	−23.38
5	190	490	0.0001498	0.4462	−53.99	−0.05399	4.462
6	85	265	0.005935	−2.338	266.8	0.2668	−23.38
7	200	500	0.0002454	0.3559	−43.35	−0.04335	3.559
8	99	265	0.005935	−2.338	266.8	0.2668	−23.38
9	130	440	0.0006121	−0.0182	14.23	0.01423	−0.1817
10	200	490	0.0000416	0.5084	−61.13	−0.06113	5.084

**Table 4:** Optimal dispatch for the 10 generating units power system.

Units	Algorithms				
	IPOA	POA	HHO	SSA	WOA
$P_{1}$	203.5350	202.8740	211.5970	202.7439	220.4145
$P_{2}$	210.4219	210.4247	215.8357	210.9169	207.4651
$P_{3}$	200.6466	200.0152	206.2645	200.0000	224.0729
$P_{4}$	238.8801	237.3994	238.8798	239.5520	242.8912
$P_{5}$	185.0712	194.4705	215.2235	190.0000	200.0707
$P_{6}$	236.0326	238.9872	238.5807	238.3172	235.6278
$P_{7}$	273.2652	269.0280	267.1062	282.0928	226.3169
$P_{8}$	238.3423	238.6122	245.7352	237.8052	239.6864
$P_{9}$	423.9302	418.0496	405.2114	408.7595	413.4965
$P_{10}$	489.8126	489.9749	454.6139	489.8125	489.9581

**Table 5:** Fuel cost ($/h) for the 10 generating unit power system.

Algorithms	Statistics			
	Min Cost	Max Cost	Mean Cost	SD
IPOA	**651.8784**	655.5161	652.6444	**1.0014**
POA	652.0687	654.4392	659.458	1.7685
HHO	653.4787	662.7219	679.2167	6.3263
SSA	651.9516	653.2228	656.5612	1.614
WOA	653.7402	672.8395	699.5087	12.5738

**Table 6:** Unit data for the 40 generating units power system with VPE.

Units	Pmin	Pmax	a	b	c	e	f
1	36	114	0.00690	6.73	94.705	100	0.084
2	36	114	0.00690	6.73	94.705	100	0.084
3	60	120	0.02028	7.07	309.540	100	0.084
4	80	190	0.00942	8.18	369.030	150	0.063
5	47	97	0.01140	5.35	148.890	120	0.077
6	68	140	0.01142	8.05	222.330	100	0.084
7	110	300	0.00357	8.03	287.710	200	0.042
8	135	300	0.00492	6.99	391.980	200	0.042
9	135	300	0.00573	6.60	455.760	200	0.042
10	130	300	0.00605	12.90	722.820	200	0.042
11	94	375	0.00515	12.90	635.200	200	0.042
12	94	375	0.00569	12.80	654.690	200	0.042
13	125	500	0.00421	12.50	913.400	300	0.035
14	125	500	0.00752	8.84	1760.400	300	0.035
15	125	500	0.00708	9.15	1728.300	300	0.035
16	125	500	0.00708	9.15	1728.300	300	0.035
17	220	500	0.00313	7.97	647.850	300	0.035
18	220	500	0.00313	7.95	649.690	300	0.035
19	242	550	0.00313	7.97	647.830	300	0.035
20	242	550	0.00313	7.97	647.810	300	0.035
21	254	550	0.00298	6.63	785.960	300	0.035
22	254	550	0.00298	6.63	785.960	300	0.035
23	254	550	0.00284	6.66	794.530	300	0.035
24	254	550	0.00284	6.66	794.530	300	0.035
25	254	550	0.00277	7.10	801.320	300	0.035
26	254	550	0.00277	7.10	801.320	300	0.035
27	10	150	0.52124	3.33	1055.100	120	0.077
28	10	150	0.52124	3.33	1055.100	120	0.077
29	10	150	0.52124	3.33	1055.100	120	0.077
30	47	97	0.01140	5.35	148.890	120	0.077
31	60	190	0.00160	6.43	222.920	150	0.063
32	60	190	0.00160	6.43	222.920	150	0.063
33	60	190	0.00160	6.43	222.920	150	0.063
34	90	200	0.00010	8.95	107.870	200	0.042
35	90	200	0.00010	8.62	116.580	200	0.042
36	90	200	0.00010	8.62	116.580	200	0.042
37	25	110	0.01610	5.88	307.450	80	0.098
38	25	110	0.01610	5.88	307.450	80	0.098
39	25	110	0.01610	5.88	307.450	80	0.098
40	242	550	0.00313	7.97	647.830	300	0.035

**Table 7:** Optimal dispatch of IPOA for the 40 generating units power system.

Units	Outputs	Unit	Outputs	Unit	Outputs	Unit	Outputs
$P_{1}$	113.1496	$P_{11}$	243.6059	$P_{21}$	523.2740	$P_{31}$	190.0000
$P_{2}$	114.0000	$P_{12}$	94.00949	$P_{22}$	523.2890	$P_{32}$	190.0000
$P_{3}$	97.40526	$P_{13}$	304.5174	$P_{23}$	523.2808	$P_{33}$	190.0000
$P_{4}$	179.7357	$P_{14}$	304.5203	$P_{24}$	523.2905	$P_{34}$	200.0000
$P_{5}$	94.50869	$P_{15}$	304.5219	$P_{25}$	523.2831	$P_{35}$	167.4762
$P_{6}$	140.0000	$P_{16}$	304.5212	$P_{26}$	523.2792	$P_{36}$	200.0000
$P_{7}$	259.6008	$P_{17}$	489.2985	$P_{27}$	10.00649	$P_{37}$	110.0000
$P_{8}$	284.6023	$P_{18}$	489.2820	$P_{28}$	10.00295	$P_{38}$	110.0000
$P_{9}$	284.6312	$P_{19}$	511.2877	$P_{29}$	10.00000	$P_{39}$	110.0000
$P_{10}$	130.0066	$P_{20}$	511.2906	$P_{30}$	97.00000	$P_{40}$	511.2834

**Table 8:** Fuel cost ($/h) for the 40 generating unit power system.

Algorithms	Statistics			
	Min Cost	Max Cost	Mean Cost	SD
IPOA	**121,591.3068**	123,933.37	122,659.9709	**654.9886**
POA	124,907.4276	129,260.1887	126,473.6095	937.3753
HHO	123,387.6705	128,381.2468	125,532.5618	1075.9279
SSA	122,693.0062	127,500.1279	124,321.0393	1124.6677
WOA	125,880.0464	134,779.6761	129,308.2354	1817.6021

**Table 9:** Fuel cost ($/h) for the 80 generating unit power system.

Algorithms	Statistics			
	Min Cost	Max Cost	Mean Cost	SD
IPOA	**244,105.2816**	249,955.5348	247,043.7003	1493.4631
POA	253,073.4467	258,114.9399	255,577.6569	1279.7300
HHO	249,554.8627	257,240.0592	252,846.8087	1948.0503
SSA	246,532.3112	251,589.9268	248,650.6662	1167.5877
WOA	258,734.1637	271,925.7694	263,327.7722	3230.8254

## Data Availability

Dataset available on request from the authors.
